# Effect of electroacupuncture on restoration of traumatic vertebral compression fracture: two case studies and literature review

**DOI:** 10.3389/fmed.2025.1612221

**Published:** 2025-08-21

**Authors:** Ji-Sung Yeum, Ye-Rim Yun, No-Hyun Kim, Jong-Ho Choi, Do-Young Kim

**Affiliations:** ^1^Department of Acupuncture and Moxibustion, Jaseng Korean Medicine Hospital, Seoul, Republic of Korea; ^2^Department of Public Health Science, Seoul National University, Seoul, Republic of Korea

**Keywords:** vertebral compression fractures, electroacupuncture, pain relief, gait disturbance, deep paraspinal muscles

## Abstract

Vertebral compression fractures (VCFs) cause severe pain and functional impairments. Conventional treatments, including medication and vertebral augmentation, have limited efficacy and safety. Electroacupuncture (EA), which combines acupuncture with electrical stimulation, is a promising but under-studied approach for VCF management. This case report with a literature review presents two cases of traumatic VCF that were successfully treated with EA as well as a literature review on this topic. Two female patients with acute VCFs were assessed using the Numerical Rating Scale (NRS), Oswestry Disability Index (ODI), and EuroQol-5 Dimension (EQ-5D) at baseline and at 2 and 4 weeks post-treatment. EA was applied to the deep paraspinal muscles using stainless steel needles (0.30 × 60 mm) inserted up to 50 mm, with 4-Hz, 1–2-mA stimulation for 20 min, twice daily. Adjunctive treatments included analgesics, herbal medicines, and thermotherapy. A literature review was conducted to determine the efficacy of EA in fracture recovery. Both patients experienced marked pain relief and functional improvements. Their NRS scores decreased from 70 to 40 (patient 1) and 30 (patient 2), ODI scores decreased from ∼85 to 64.4 and 42.2, respectively, and EQ-5D scores improved to 67.7 (patient 1) and 73.0 (patient 2). Early restoration of standing ability and ambulation was also observed. The findings from the literature review suggest that EA may enhance bone healing via callus formation and immune modulation. EA applied to the deep paraspinal muscles may be an effective, minimally invasive treatment for VCF, promoting pain relief and recovery. Further controlled trials are required to confirm the efficacy and mechanism of action.

## 1 Introduction

Vertebral compression fractures (VCFs) are spinal injuries resulting from trauma or bone fragility (1). In 2019, approximately 8.6 million new cases of spinal fractures were reported globally, reflecting a 37.7% increase since 1990 (2, 3). Patients with VCFs commonly present with severe pain during postural changes, coughing, or lifting, and in severe cases, neuropathies, such as muscle weakness and sensory deficits (4). Patients with VCFs experience a significantly reduced quality of life (QoL) owing to physical disability, psychological distress, and delayed social reintegration (5, 6). In the United States, the annual economic burden of VCFs is estimated to be $746 million (6).

Bed rest is commonly recommended for the treatment of VCFs; however, early intervention is crucial for pain relief, quick recovery, and minimization of complications (7). Current clinical guidelines recommend vertebral augmentation as a surgical treatment; however, its efficacy and safety remain controversial (8). Studies have suggested that this procedure does not lead to significant improvements in pain relief, physical function, QoL, or perceived recovery compared to placebo treatments (9). Additionally, it poses a high risk of complications, such as nerve damage and infection due to cement leakage (6, 8). Consequently, the interest in conservative treatments for VCF, including physical therapy, acupuncture, and pharmacological interventions, is increasing (10).

Electroacupuncture (EA), a minimally invasive technique that combines acupuncture with electrical stimulation, is widely used to treat various neuromuscular disorders, including arthritis, sprains, Bell’s palsy, and spinal cord injury (11, 12). In fracture management, EA has been found to enhance bone restoration by accelerating callus formation and bone mineralization (13). Furthermore, EA activates the nerves and muscles through electrical currents, facilitating the release of neurotransmitters and growth factors to accelerate tissue repair (14). Given the anatomical characteristics of the spine, recovery after a VCF is often delayed because of limited peripheral circulation to damaged tissues (15). While evidence on the effectiveness of EA in treating VCFs is limited, some studies speculate that it promotes blood flow, reduces inflammation, enhances tissue regeneration, and restores function (16).

Previous studies have focused on acupuncture or physical therapies for VCFs, but not EA targeting deep spinal structures. This report presents cases of two patients with VCFs who were treated with EA applied to the deep paraspinal soft tissues.

## 2 Case description

### 2.1 Clinical presentation and diagnosis

We present the cases of two patients who received inpatient care at the Jaseng Hospital of Korean Medicine for the chief complaint of severe spinal pain without radiative leg pain owing to traumatic VCFs caused by a traffic accident. Both patients were admitted via emergency ambulance and were unable to stand or move upon arrival, but had normal vital signs and no alterations in consciousness. Open wounds were not observed. The initial physical examination revealed swelling, tenderness, and localized heat in the thoracolumbar region, with no sensory deficits or muscle weakness.

**FIGURE 1:**
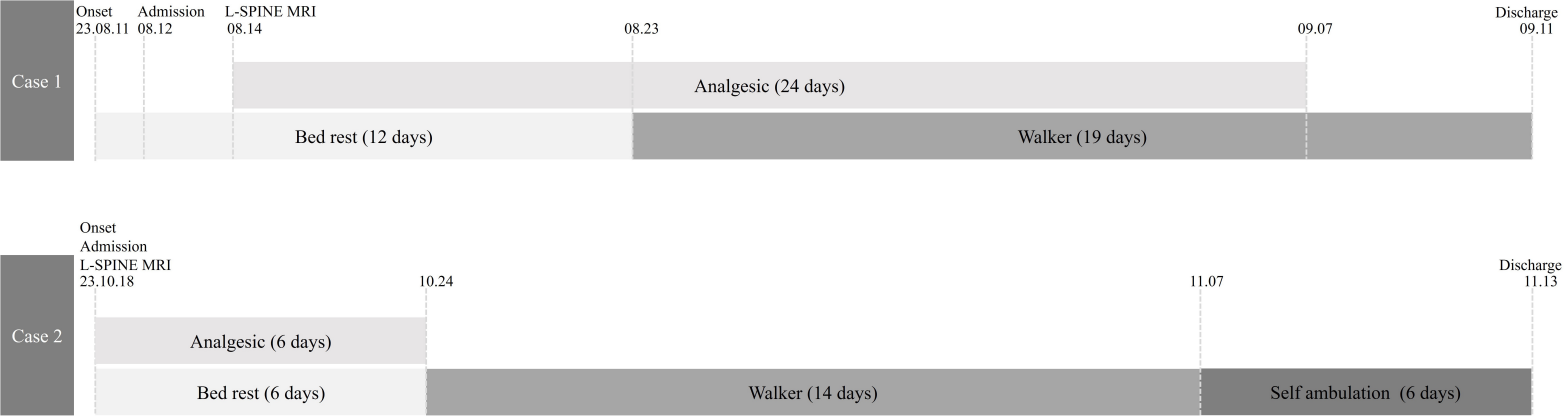
Timeline of the cases. In the horizontal axis, the upper bar represents the status of analgesic medication use and the lower bar, orthostatic and gait abilities. MRI, magnetic resonance imaging.

**FIGURE 2:**
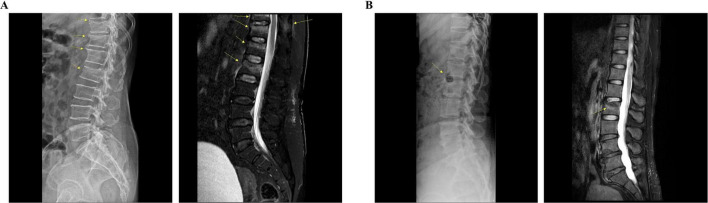
Radiological findings of each patient. **(A)** Patient 1: lateral radiograph reveals subtle bone deformities, including radiolucent lines, extending from T11 to L2. Sagittal T2-weighted fat-saturated MRI shows low signal intensity lines with bone marrow edema in the T12, L1, and L2 vertebral bodies, indicative of recent fractures. Additionally, a recent fracture can be observed at the T11 vertebral body, accompanied by bone marrow edema in the spinous process. **(B)** Patient 2: lateral radiograph reveals bone lesions, including a hypo-dense lesion in the anterior column of the L2 vertebra. Sagittal T2-weighted fat-saturated MRI demonstrates a compression fracture with bone marrow edema in the L2 vertebral body. MRI, magnetic resonance imaging.

The diagnosis of VCF was confirmed via radiography and magnetic resonance imaging (MRI) during the admission period (Figures 1, 2). Patient 1 was a 68-year-old woman with a history of hyperlipidemia, hypertensive heart disease, and osteoporosis. Radiography and MRI confirmed recent compression fractures at the T11–L2 vertebral body (Figure 2A). She reported severe thoracolumbar pain radiating to the chest, which was exacerbated by coughing and frequent sleep disruption. Meanwhile, Patient 2 was a 49-year-old woman without any medical history; she presented with acute L2 vertebral body compression fracture (Figure 2B). She complained of severe back pain, nausea, and diarrhea, which disrupted her sleep. Both patients had unremarkable psychosocial and genetic histories. Other than a one-time administration of analgesics following the onset of symptoms, discontinued upon hospital admission, there were no relevant prior interventions.

This study was approved by the Institutional Review Board (IRB) of Jaseng Hospital of Korean Medicine (approval no. JASENG 2024-12-008; approval date: December 18, 2024).

### 2.2 Measurement and treatment

Patient-reported outcome measures were used to assess pain severity, spinal disorder-related disabilities, and QoL. Pain severity was evaluated using a Numerical Rating Scale (NRS), while the EuroQol-5 Dimension (EQ-5D) (17) and Oswestry Disability Index (ODI) (18) were used to assess QoL and disability, respectively. Assessments were conducted at baseline (week 0) and at 2 and 4 weeks post-treatment; the scores were converted using a 0–100 scale (higher scores indicated worse NRS and ODI but better EQ-5D).

The patients were initially placed on bed rest because of the inability to move and risk of exacerbating fractures. Conservative treatment was initiated on the first day of hospitalization, and EA was implemented as the primary treatment. For this procedure, a stainless steel acupuncture needle (0.30 × 60 mm; Dongbang Acupuncture Inc., Seoul, South Korea) was inserted to a depth of 50 mm to reach the deep paraspinal muscles or up to the adjacent laminar muscles, and an electrical stimulator (STN-110; Stratek Co., Anyang, Korea), with a continuous wave pattern for 20 min at a frequency of 4 Hz and intensity of 1–2 mA, was used. Additional therapies, including analgesics, herbal medicines, and thermotherapies, were administered to alleviate pain and promote peripheral circulation. Treatment was administered twice daily during hospitalization. Ambulation with a spinal brace and walker was initiated only under medical supervision and strictly limited to pain-free, minimal movements. Details of the treatment protocol are summarized in Supplementary Table 1, and the treatment timelines for both cases are illustrated in Figure 1.

### 2.3 Clinical course and outcomes

**FIGURE 3:**
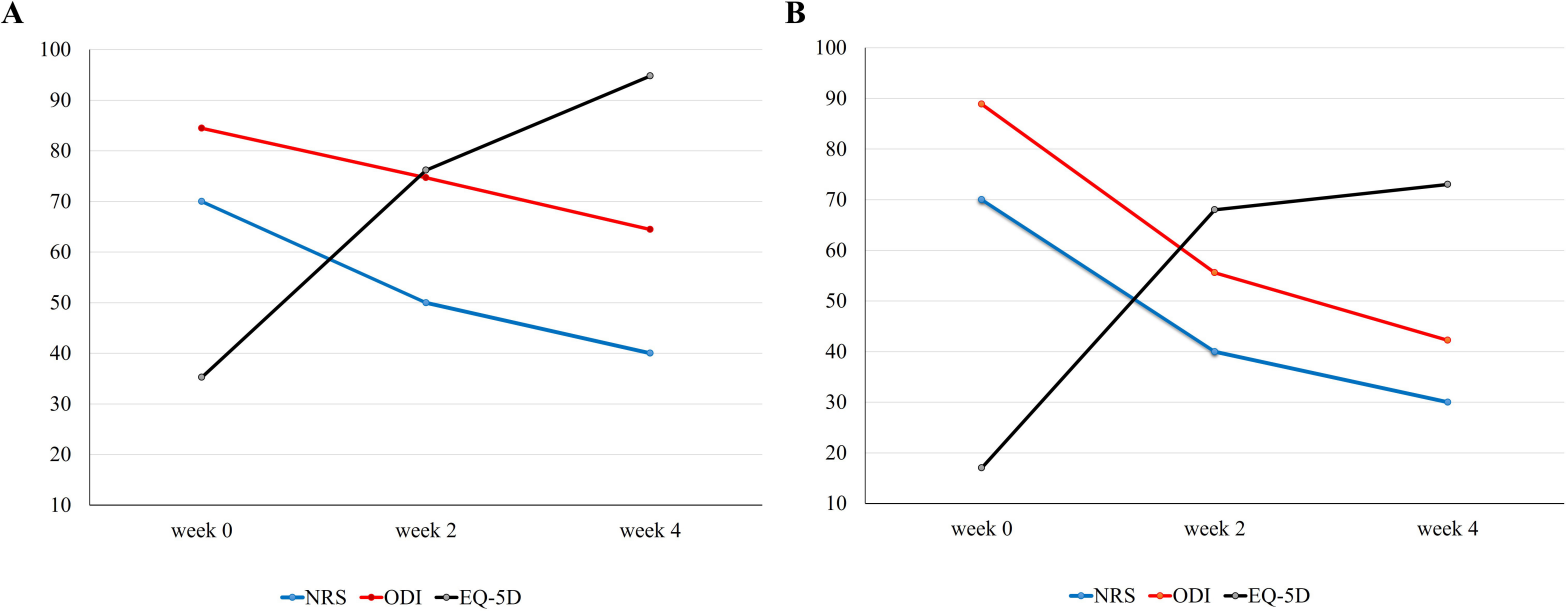
Course of symptoms. Changes in symptoms [**(A)** patient 1, **(B)** patient 2]: low back pain, physical disability scores, and quality of life were measured using the NRS, ODI, and EQ-5D, respectively. EQ-5D, EuroQol-5 Dimension; NRS, Numerical Rating Scale, ODI: Oswestry Disability Index.

At baseline, both patients presented severe functional impairment and pain, with NRS and ODI scores of 70 and 85 for Patient 1, and 70 and 90 for Patient 2, respectively. Pain, function, and QoL steadily improved during treatment as assessed at 2 and 4 weeks. By the time of discharge, both patients had achieved notable recovery and were able to maintain basic daily routines independently. The EQ-5D score of Patient 1 improved to 67.7, ODI decreased to 64.4, and NRS decreased to 40. Patient 2 demonstrated similar progress, with an EQ-5D score of 73.0, ODI of 42.2, and NRS of 30 (Figure 3). Both patients showed early restoration of standing and gait functions with brace. Patient 1 could stand without pain by day 10 of hospitalization, and Patient 2, by day 5. Gait training with a walker was introduced the following day for both patients (Figure 1). At discharge, both the patients walked at a normal pace without assistance. During the entire course of treatment, no adverse events or side effects were observed in these cases.

### 2.4 Literature review

A literature search was conducted using PubMed to identify studies on EA interventions for the treatment of bone fractures. The inclusion criteria were as follows: (1) studies involving human or animal subjects with fractures, (2) clinical or mechanistic studies evaluating the effects of EA, and (3) studies that conducted statistical analysis of the effectiveness of EA vs the control condition. The primary search terms included “fracture” and “electroacupuncture,” and articles were screened by reading of the titles, abstracts, and full texts.

Of the 47 initially identified articles, one randomized controlled trial (RCT) and two animal studies met the inclusion criteria. The characteristics of these studies are summarized in Table 1. The two animal studies suggested that EA significantly promoted bone recovery by enhancing callus formation and modulating immune responses within the fracture site (13, 19). The RCT demonstrated a statistically significant improvement in pain and physical function in the EA group compared with the exercise group (20).

**TABLE 1:** Characteristics of the studies.

References	Study design (type of Fx)	Intervention	Control	Findings
Nakajima et al. (13)	Animal study (tibial Fx.)	50 Hz, 20 μA, 20 min daily for 3 weeks	Sham EA No treatment	Formation of callus: *P* < 0.01 Strength of callus: *P* < 0.001
Inoue et al. (19)	Animal study (fibular Fx.)	50 Hz, 20 μA, 20 min 5 d a week for 6 weeks	No treatment	Bone defect: *P* < 0.001 Immune cell count: *P* < 0.05
Tian et al. (20)	RCT (after tibial Fx. surgery)	30 min daily for 2 weeks*	Exercise	Pain: *P* < 0.01 Physical function: *P* < 0.001

*Information on the frequency and intensity of EA is not available in the original article. EA, electroacupuncture; Fx, fracture; RCT, randomized controlled trial.

## 3 Discussion

With the global increase in life expectancy, the management of traumatic VCFs caused by external forces, such as falls, has emerged as a significant healthcare issue in the older population (21). According to the published clinical guidelines, no pharmacological or surgical treatment for VCFs is currently supported by strong evidence (8). Given the demographic characteristics of patients with VCFs, minimally invasive interventions are preferred, highlighting the need for novel therapeutic strategies to bridge the gap between conservative treatments and surgery (22). Evidence on the efficacy of EA in treating VCFs remains limited. In this study, we report the cases of two patients with traumatic VCF successfully treated with EA; EA promoted pain relief and functional restoration. We also conducted a literature review to explore its potential for further clinical applications.

As summarized in Figure 1, both patients were initially on bed rest, followed by gait training with a supporting device as soon as possible. Analgesic medication was administered alongside conservative therapies and prospectively planned to maintain sleep quality, which is a crucial factor in bone mineral density and fracture recovery (23). With continuous treatment, both patients were able to stand and walk using a walker in the second week (Figure 1). However, the improvement in NRS and ODI was greater in Patient 2, indicating better prognosis (Figure 3). As expected, Patient 1 had a higher number of fractured vertebral bodies, including spinous process fractures, and more severe vertebral collapse, leading to a longer bed rest period and greater analgesic use than Patient 2 (Figures 1, 2). A multicenter cohort study reported that patients with acute vertebral fractures who received only bracing without any other treatment required up to 12 weeks to ensure full pain relief and QoL improvement (24). In this context, the return to daily activities after approximately 4 weeks of treatment in both our patients suggests a relatively earlier recovery of pain and function than the expected course of VCF healing.

By needling the deep paraspinal muscles with electrical stimulation, EA may provide rapid pain relief and promote tissue restoration in patients with VCF. The analgesic effects of EA are well documented, with evidence suggesting that needling plays a crucial role (25). Manual acupuncture not only modulates inflammatory reactions but also activates afferent fibers, leading to the modulation of various signaling molecules, including opioid peptides, glutamate, and serotonin (26). Electrical stimulation enhances these effects and further promotes the release of enkephalin and dynorphin within the central nervous system (25). Although the 2010 American Academy of Orthopedic Surgeons guidelines recommended electronic physical therapy for VCF-related pain, its efficacy may be limited because of its transcutaneous nature, unlike EA (8). Our case studies suggest that the ability of EA to deliver electrical stimulation in close proximity to pathological lesions in a minimally invasive manner, in addition to needling, may be a promising mechanism underlying its analgesic effects.

Regarding the promotion of tissue restoration by EA, evidence indicates that electrical stimulation enhances wound healing in the bone, muscles, tendons, and skin (27). This mechanism was supported by our literature review (Table 1). Two preclinical studies using rodent models of tibial or fibular fractures demonstrated effective bone repair, evidenced by immune responses and callus formation (13, 19). Electrical stimulation enhances peripheral circulation by increasing blood supply and upregulating tissue restoration factors, such as bone morphogenetic proteins and growth factors (28). Its bone-healing effects are further supported by studies using electronic bone growth stimulators, which showed efficacy *in vitro* and in small animal models (29). However, these effects were not replicated in large animal studies, due to limitations in the penetration of non-invasive modalities. In our patients, electrical stimulation delivered via needles to deep paraspinal tissues may have facilitated VCF recovery. However, it is important to note that none of the reviewed studies specifically modeled VCFs, and thus the mechanisms may not fully generalize to our cases. Additionally, adjunctive therapies, including thermotherapy, herbal medicines, and natural products promoting peripheral circulation, may have had synergistic effects, (30, 31) (Supplementary Table 1). Therefore, the effects observed cannot be attributed solely to EA.

The primary targets of EA in this study were the deep paraspinal muscles, including the erector spinae and transversospinalis, which are crucial for spinal alignment, postural balance, and gait (32). Impairment of these muscles disrupts spinal mechanics, leading not only to gait disturbances and delayed VCF recovery but also to an increased risk of VCF recurrence (32, 33). Notably, our patients were able to stand independently and walk with a brace within 1–2 weeks (Figure 1); therefore, EA may support muscle coordination and facilitate functional recovery, thereby improving gait disturbances. Because low-intensity exercise is a key conservative and preventive strategy for VCF, concurrent gait training with assistive devices may have further accelerated recovery (34). Future myo-neuronal studies in patients with VCFs are warranted to investigate the effects of EA targeting deep paraspinal tissues on spinal integrity and gait function.

This study had some limitations. The concurrent application of other conservative treatments along with EA may have had synergistic therapeutic effects. Therefore, further controlled studies are required to determine the specific effects of EA. Additionally, the lack of follow-up data such as MRI data, gait distance or postural pictures after discharge prevented the assessment of long-term therapeutic outcomes, potentially introducing biases, such as the Hawthorne effect. Finally, assessments of symptom improvement from small sample size were mainly based on patient-reported outcomes, which are inherently subjective. Objective measures, such as follow-up MRI or blood biomarkers, would have allowed for a more comprehensive analysis. Nevertheless, to our knowledge, we present the first case series in which EA targeting the deep paraspinal muscles was successfully employed as the primary treatment for traumatic VCFs. As interest in non-pharmacologic and minimally invasive interventions grows, these findings may help guide future research. Well-designed, large-scale clinical trials investigating EA as a standalone treatment, particularly with medium- to long-term follow-up assessing both pain and functional outcomes, are warranted. In addition, VCF-specific mechanistic studies on bone healing are necessary to elucidate the biological basis of EA’s therapeutic potential.

## 4 Conclusion

We report two cases of VCF that were successfully treated with EA targeting the deep paraspinal muscles that support spinal alignment, postural balance, and gait stability. EA may promote rapid pain relief by stimulating analgesic substance release and enhanced tissue repair through improved peripheral flow and growth factor upregulation, with minimal side effects. These findings highlight the potential of EA as a minimally invasive therapy for VCF that can promote quick recovery.

## Data Availability

The original contributions presented in this study are included in this article/Supplementary material, further inquiries can be directed to the corresponding author.
