# Supplementary material for: Effect of electroacupuncture on restoration of traumatic vertebral compression fracture: two case studies and literature review
Source: Front Med (Lausanne). 2025 Aug 21;12:1612221. doi: 10.3389/fmed.2025.1612221 (PMC12408302; doi:10.3389/fmed.2025.1612221)
Supplement: Supplementary file 1 [file Table_1.docx]

| **Supplementary Table 1. Contents of conservative treatment** | |
| --- | --- |
| Intervention | Details |
| Electroacupuncture | - Target tissues were erector spinae (iliocostalis, longissimus, spinalis), multifidus muscles, and iliolumbar ligament - Perpendicular insertion of needle (40–50-mm depth) with 1–2-mA, 4-Hz of electronic stimulation for 20 min |
| Pharmacopuncture | - Shinbaro2^A^ intramuscular injections on tender points |
| Dry needling | - Tender points (myofascial trigger points) |
| Herbal medicine | - Hwalhyeoljeongtong-tang (Patient 1)   *Saposhnikovia divaricata, Paeonia lactiflora, Angelica gigas, Prunus persica, Glycyrrhiza uralensis, Schizonepeta tenuifolia, Citrus reticulata, Akebia quinata, Commiphora myrrha, Lindera strychnifolia, Dipsacus asper, Boswellia carterii*   - Hwalhyeoljitong-tang (Patient 2)   *Poria cocos, Ligusticum chuanxiong, Angelica sinensis, Glycyrrhiza uralensis, Paeonia lactiflora, Citrus reticulata, Carthamus tinctorius, Commiphora wightii, Caesalpinia sappan, Boswellia sacra* |
| Analgesic | - Naproxen, 500 mg 2T #2 (Patient 1) - Loxoprofen, 60 mg 2T #2 (Patient 2) |
| Other treatments | - Moxibustion - Cupping therapy - Infrared therapy |
| ^A^Hong et al., Repeated epidural delivery of Shinbaro2: effects on neural recovery, inflammation, and pain modulation in a rat model of lumbar spinal stenosis. *Front Pharmacol*. 2024;15:1324251. | |
